# Experimental Hybrids of the *Triatoma brasiliensis* Species Complex Show Higher Susceptibility to the *Trypanosoma cruzi* Infection Than Their Parentals

**DOI:** 10.3390/microorganisms11122850

**Published:** 2023-11-24

**Authors:** Nathália Correia, Letícia Paschoaletto, Carolina Reigada, Teresa Cristina Monte Gonçalves, Carlos José de Carvalho Moreira, Jane Costa

**Affiliations:** 1 Entomology Laboratory, Oswaldo Cruz Institute, Fiocruz, Rio de Janeiro 21040-360, Brazil; nathcor@gmail.com; 2 Molecular Parasitology Laboratory, Federal University of Rio de Janeiro, Rio de Janeiro 21941-901, Brazil; paschoaletto@biof.ufrj.br; 3 Center of Biological and Health Sciences, Department of Ecology and Evolutionary Biology, São Carlos University, UFSCAR, São Carlos 13565-905, Brazil; creigada@ufscar.br; 4 Interdisciplinary Laboratory for Entomological Surveillance of Diptera and Hemiptera, Oswaldo Cruz Institute, Fiocruz, Rio de Janeiro 21040-360, Brazil; tcmontegoncalves@gmail.com; 5 Parasitic Diseases Laboratory, Oswaldo Cruz Institute, Fiocruz, Rio de Janeiro 21040-360, Brazil; cjcmoreira@gmail.com; 6 National and International Reference Laboratory in Taxonomy of Triatomines, Oswaldo Cruz Institute, Fiocruz, Rio de Janeiro 21040-360, Brazil

**Keywords:** Chagas disease, triatomine, hybrid, vector capacity, metacyclogenesis, epimastigotes

## Abstract

The *Triatoma brasiliensis* species complex is a monophyletic group encompassing two subspecies and six species. Recently, a hybrid zone of members of this complex was recorded in the state of Pernambuco. Questions concerning the capability of the hybrids to become infected with *Trypanosoma cruzi* have been raised. This study aimed to compare the susceptibility of *Triatoma b. brasiliensis*, *Triatoma juazeirensis*, and their experimental hybrids to infection with *T. cruzi*. We infected the parentals and their experimental hybrids (obtained through reciprocal crosses) through artificial feeding with citrated rabbit blood, to which the TcI 0354 strain of *T. cruzi* had been added. The insects were weighed before and after feeding on the rabbit blood, and then they were dissected on the 10th, 20th, and 30th day after infection. Both the hybrids and the parentals remained infected throughout the experiment. The parasite was mostly found in the epimastigote form. The number of epimastigotes was significantly lower in the stomach and small intestine of *T. juazeirensis* than in the hybrids or in *T. b. brasiliensis*. A significantly higher percentage of metacyclic trypomastigotes was detected in the small intestine and rectum of the hybrids. Hybrids demonstrated higher susceptibility to the TcI 0354 strain than their parentals, opening up new avenues to be investigated.

## 1. Introduction

Chagas disease remains one of the most important and yet neglected diseases in the world, and until now, there have been no drugs available to cure the illness in its chronic phase [1]. Therefore, strategies that aim to monitor and control insect vectors are the most effective measures to prevent the transmission of the protozoan *Trypanosoma cruzi,* an etiologic agent, to humans [2]. American trypanosomiasis, better known as Chagas disease, affects more than six million people around the world, and changes in its epidemiology pose new challenges for controlling the illness [2,3]. Currently, there are more than 155 triatomine species recognized as potential vectors of *T. cruzi* that occur mainly in Central and South America; however, only a dozen of them offer a real risk for the transmission of this etiological agent to human populations [4,5,6,7,8].

*Trypanosoma cruzi* is a flagellated protozoan parasite of mammals that is transmitted via bloodsucking insects of the Triatominae subfamily. It causes Chagas disease, an important human disease in Neotropical America [4,9,10]. Distinct methods of transmission of the protozoan have also been reported among human populations, for instance, through the consumption of contaminated wild animals [11,12,13]. The disease is now considered a global public health problem due to human migration from endemic to non-endemic areas, where countries such as Australia, Canada, Japan, Spain, and the United States of America have the highest number of immigrants infected by *T. cruzi* [14].

The parasite develops inside the triatomine insect’s intestinal tract, and its first transformation from trypomastigotes to epimastigotes takes place in the stomach, where the blood is stored nearly undigested. When the parasite reaches the small intestine, a multiplication boost occurs, and the parasite population density increases. Some of the parasites migrate to the rectum, while others continue to multiply in this portion of the intestinal tract. The different properties of the environment of the rectum provide better attachment conditions for the parasite, which leads to a transformation in its infective form, i.e., the metacyclic trypomastigote form [15]. Subsequently, the insects eliminate the parasite’s infective form in their feces and urine, which can be deposited on the skin of the mammal host species, eventually transmitting *T. cruzi* [12,16,17]. The triatomines’ vector capacity is related to several factors: their geographic distribution, feeding behavior, and physiological, genetic, and environmental parameters [17,18,19,20].

The *Triatoma brasiliensis* species complex is a monophyletic group of the Triatominae subfamily, and multidisciplinary studies have been carried out to reveal the phylogenetic relationships among the species [21,22,23,24,25,26]. Currently, this complex encompasses two subspecies and six species: *T. b. brasiliensis* Neiva, 1911; *T. b. macromelasoma* Galvão, 1956; *T. bahiensis* Sherlock & Serafim, 1987; *T. juazeirensis* Costa & Felix, 2006; *T. lenti* Sherlock & Serafim, 1967; *T. melanica* Neiva e Lent, 1941; *T. petrocchiae* Pinto & Barreto, 1925; and *T. sherlocki* Papa et al., 2002. The members of this species complex are of distinct epidemiologic importance, have a clear geographic distribution, and can be distinguished by their morphological characteristics [22,27,28,29,30]. *Triatoma b. brasiliensis* is one of the most important triatomine species in northeastern Brazil because of its wide geographic distribution (in five Brazilian states), its high rate of domiciliation, and its spread through natural infection [27,29,31,32]. Moreover, the programs to control *Triatoma infestans* (Klug, 1834), which was previously considered the main vector in Latin America, were not so effective for *T. b. brasiliensis*, a native vector, which colonized not just human domiciles but also infested several artificial and natural ecotopes [6,27,31,32,33,34,35,36,37].

Morphometric studies on the *T. brasiliensis* species complex have identified *T. b. macromelasoma* as a possible hybridization product between *T. juazeirensis* and *T. b. brasiliensis*, suggesting, for the first time, a homoploidal hybrid speciation in the triatomine group [38]. In addition, thirteen different phenotypes of *T. b. brasiliensis* were found in peridomiciliary areas in the state of Pernambuco, and their intermediate patterns were confirmed using molecular tools, establishing them as hybrids of members of this species complex [39].

The triatomine’s susceptibility to infection depends on several factors, such as the vector species becoming infected by the parasite; the parasite density, which is modulated by the insect’s physiological barriers [15,40]; the multiplication rates of the parasite; the capacity of the parasite to reach the insect’s rectum [41]; and adaptation of the parasite strain to the triatomine species [40,42,43]. On the other hand, *T. cruzi* strains have biological, biochemical, molecular, and genetic diversity, along with eco-epidemiological complexity [44,45]. Therefore, interactions between the parasite and insect vectors raise complex questions that are yet to be understood.

Studies on the capacity of the triatomine species to become infected with the parasite *T. cruzi* and how this is associated with its capacity to colonize human domiciles are of great importance for public health and governmental services since this information is crucial for the application of more precise monitoring and measures to control the Chagas disease vectors [2,27,46].

The objective of this study was to explore the susceptibility of the hybrids from *T. b. brasiliensis* and *T. juazeirensis* to *T. cruzi* in comparison to the susceptibility of their parental subspecies and species by performing experimental infection.

## 2. Materials and Methods

### 2.1. Insects

*Triatoma b. brasiliensis* and *T. juazeirensis* fifth-instar nymphs from laboratory colonies kept under standardized conditions (52–70% relative humidity and 23–24.8 °C) were randomly selected (Table 1).

Initially, 10 fifth-instar nymphs (5 males and 5 females) from each species were sexed and separated into containers (14 × 14 × 15 cm) to obtain virgin adults. The insects were fed once a week on Swiss Webster mice (license: LW-18/11 from the Ethics Committee on the Use of Animals of the Oswaldo Cruz Institute (CEUA-IOC)) until reaching the imaginal molt. When some of the specimens died, they were replaced by other virgin adults. Thirty other fifth-instar nymphs of *T. b. brasiliensis* and *T. juazeirensis* were kept starving for 30 days and were then used in the infection experiment.

### 2.2. Experiments of Species Crossing

After the imaginal molt, interspecies crosses of *T. b. brasiliensis* females × *T. juazeirensis* males and *T. juazeirensis* females × *T. b. brasiliensis* males were performed in separate containers, and they were fed mice once a week. The hybrids were named “Hbj” and “Hjb”.

**Figure 1:**
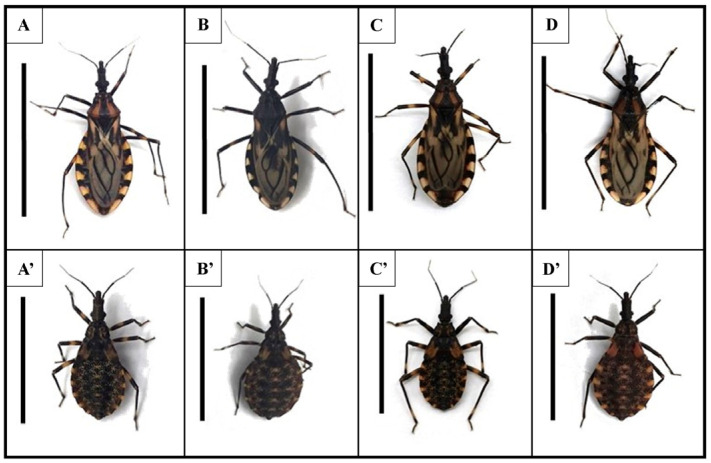
General aspect of adults (**top**) and fifth-instar nymphs (**bottom**) of *Triatoma brasiliensis* (**A**,**A’**), *Triatoma juazeirensis* (**B**,**B’**), and their reciprocal hybrids “Hbj” (♀ *T. b. brasiliensis* × ♂ *T. juazeirensis*) (**C**,**C’**) and “Hjb” (♀ *T. juazeirensis* × ♂ *T. b. brasiliensis*) (**D**,**D’**), scale= 25mm for adults and 20 mm for nymphs.

The F1 hybrids, thus obtained, were placed in separate containers and were reared until at least 30 fifth-instar nymphs had been obtained from each crossing. In total, 30 fifth-instar specimens of *T. b. brasiliensis* and *T. juazeirensis* plus 60 F1 hybrids (30) of each crossing combination were used in the infection experiment with strain 0354 of *T. cruzi (*Figure 1).

### 2.3. Trypanosoma cruzi

The parasite isolate used in this study was obtained from the institutional trypanosome collection consisting of wild and domestic mammals and vectors (*Coleção de Trypanosoma de Mamíferos Silvestres*, *Domésticos e Vetores*, COLTRYP). *Trypanosoma cruzi* strain 0354 (TcI) was originally isolated from *T. b. brasiliensis* from Caicó, its type locality, and it has been maintained through cryopreservation at −195 °C in liquid nitrogen since 2006. This isolate was previously characterized by means of multiplex PCR on the mini-exon gene, as described by another study [47].

Epimastigotes were grown in MacNeal, Novy, and Nicolle (NNN) medium with a liver infusion tryptose (LIT) overlay and supplemented with 10% fetal bovine serum, as previously described in [40,48].

### 2.4. Infective Feeding of the Insects

Thirty fifth-instar nymphs of the parental species (*T. b. brasiliensis* and *T. juazeirensis*) and their hybrids (Hbj and Hjb) were artificially fed through latex membranes 10 mL of citrated rabbit blood (0.1 mL of sodium citrate/mL), which had been centrifuged at a speed of 3500 rpm for 10 min to separate the plasma from the erythrocytes. We removed the plasma and washed the erythrocytes three times with phosphate-buffered saline (PBS; pH 7.2).

After washing, the erythrocytes were resuspended in LIT (the same volume as that of the plasma removed) containing 1.5 × 10^7^/mL epimastigotes in the exponential growth phase (*v*/*v*), and they were counted using a hemocytometer. This mixture was then transferred to a glass bottle where peripheral circulation of heated water was carried out at a temperature of around 37 °C. A latex membrane coated the open bottom of the bottle, on which the insects fed. Fifteen insects were divided between two bottles so that all of the insects could move and reach the food source. Thus, only fully engorged insects were used in the experiment.

For this experiment, the insects were previously and individually marked with different gouache colors (non-toxic) on the legs and pronotum, allowing for the differentiation of each of the specimens. Then, we weighed the insects individually on a precision scale (Libor AEG, Shimadzu, Kyoto, Japan) before and after the infective meal. Thus, it was possible to measure their blood volume ingestion by calculating the weight difference, which was based on the weight before and after the blood meal (Supplementary Table S1).

Fifteen days after the first feeding, the insects were fed again with chicken blood without parasites because the percentage of flagella declines if the insects are subjected to long periods of fasting [15].

### 2.5. Analysis of T. cruzi Forms in Insects

Ten triatomines were dissected 10, 20, and 30 days after the infective blood meal, totaling thirty insects in each group. The outer edge of the abdomen of the triatomine (conexivum) was cut in the posterior–anterior direction and, with the dorsal region exposed, the entire digestive tract was removed and transferred to a Petri dish where it was divided into three segments: stomach, intestine, and rectum. These segments were then macerated in 200 μL of PBS (pH 7.2).

To examine the insect’s biological content (feces, urine, and digestive tract tissues), each segment of the digestive tract was individually displayed on a hemocytometer to count the distinct parasite forms: epimastigotes, metacyclic forms, and transitional forms [40,42,48,49,50,51] (Supplementary Table S2). For a better understanding and visualization of the percentages in the distinct compartments of the insect’s gut, two tables are presented wherein the first shows the percentages of the different *T. cruzi* forms across the experiment and the second compares the percentages of the parasite forms in each gut compartment.

### 2.6. Statistical Analyses

The effects of the number of days after the infective blood meal and groups on the number of parasite forms in the stomach, intestine, and rectum were analyzed by fitting generalized linear models (GLM) with the Poisson error distribution, which was corrected for overdispersion. The choice of the best statistical model applied in the analysis was made through comparisons between the adjustments of the complete models and models with reduced variables, where a maximum likelihood test was used to compare changes in deviances before and after removing variables. Interactions between variables were considered only when significant. The goodness of fit was determined using “half-normal-plot” plots with simulated envelopes at a 95% significance level. For the analysis, the statistical software R version 4.1.3 (The R Foundation for Statistical Computing; http://www.R-project.org, accessed 4 May 2023) was used. When the effect of the variables was significant, the averages of the number of parasite forms were compared using the cld function of the multcomp package of the R software (R Core Team 2020).

## 3. Results

### 3.1. Percentage of the T. cruzi Forms across the Days of Analysis

The forms found in the digestive tube were classified as epimastigotes, trypomastigotes, and transition forms. The latter includes any parasite intermediate forms, as already described in the literature [40]. Table 2 shows the variation in the relative percentage of *T. cruzi* forms (per specimen, for each time) of each form found in each compartment of the digestive tube over the course of the analysis. It was verified that there was a higher percentage of epimastigote forms in all compartments of the digestive tube in both parentals and hybrids, despite their numbers decreasing between the stomach and the rectum until the end of the experiment. The highest percentages of metacyclic forms (the infective ones) in the rectum were observed in the hybrids Hbj and Hjb.

The percentage of parasite numbers in the stomach declined over the course of the analysis for both hybrids and for *T. b. brasiliensis*. *Triatoma juazeirensis* also declined, but the kinetics were different (Table 2).

In the small intestine, the parasite percentage increased in the hybrids over the days of observation. The parentals also experienced increases in parasite percentages, although *T. juazeirensis* presented with the highest percentage on the 20th day, and for *T. b. brasiliensis*, the values were very close on the 20th and 30th days (Table 2).

In general, the percentages of parasites in the rectum were lower when compared to the other parts of the digestive tube. There was also an increase in percentages of the parasites in the rectum over the course of the analysis, mainly for Hbj and *T. b. brasiliensis* (Table 2).

### 3.2. Developmental Stages in Each Compartment of the Digestive Tube

Epimastigotes were the predominant form in all compartments and in all groups, demonstrating the highest percentages across all of the days of observation (Table 3). Transitional forms, when compared to the epimastigotes, were recorded in lower percentages in all compartments of the digestive tract in all of the insects, with oscillating values in both the hybrids and the parentals (Table 3). Trypomastigotes were observed in all parts of the digestive tract of the insects, although they had lower percentages compared to the epimastigotes and demonstrated oscillating values when compared to the transitional form. Among the specimens studied, *T. b brasiliensis* and *T. juazeirensis* had the lowest numbers of trypomastigotes in their intestines (Table 3).

The number of epimastigotes (F_1,85_ = 65.6, *p* < 0.001), transition forms (F_1,85_ = 16.7, *p* < 0.001), and trypomastigotes (F_1,85_ = 11.63, *p* < 0.001) of *T. cruzi* in the insects’ stomachs differed after long periods of time. On the other hand, the number of epimastigotes (F_3,86_ = 1.43, *p* = 0.23) and transition forms (F_3,86_ = 0.67, *p* = 0.56) of *T. cruzi* was not different among the triatomine groups (parentals and hybrids). Only the number of trypomastigotes was significantly distinct among the groups (F_3,86_ = 5.83, *p* < 0.01).

The number of epimastigotes and transition forms in the small intestine varied either with time (F_1,113_ = 5.32, *p* < 0.05 and F_1,113_ = 5.62, *p* < 0.05, respectively) or based on the triatomine group (F_3,113_ = 5.53 *p* < 0.05 and F_1,113_ = 11.38, *p* < 0.05, respectively). The variation in the number of trypomastigotes in the small intestine was affected by the interaction effects of the triatomine group and time (time: F_1,113_ = 6.93 *p* < 0.05; triatomine group: F_3,114_ = 3.43, *p* < 0.05; time× triatomine group: F_3,110_ = 4.75, *p* < 0.05). In this case, the highest percentages of trypomastigotes were found in hybrids Hbj and Hjb with oscillating values across the days of observation in both groups. In the parentals, the highest values were recorded on the 30th day, being 5% and 4% for *T. juazeirensis* and *T. b. brasiliensis*, respectively (Table 3).

The number of epimastigotes and transient forms in the rectum varied only with the observation time (F_1,93_ = 6.55, *p* < 0.05 and F_1,93_ = 4.62, *p* < 0.05, respectively). However, the different groups of triatomines had no significant effect on these *T. cruzi* forms (F_3,94_ = 0.279, *p* = 0.83; F_3,94_ = 6.55, *p* = 0.348). In general, the number of these forms of *T. cruzi* increased with observation time.

In the rectum, the hybrids showed the highest percentages of trypomastigotes (16% on the 20th day in the Hbj and 31% on the 20th day in the Hjb), when compared to the parentals. In *T. juazeirensis* and *T. b. brasiliensis*, trypomastigotes were not recorded on the 10th day, while their highest percentages (6.5% and 7%, respectively) were observed on the 30th day (Table 3).

### 3.3. Blood Meal Volume

As shown in Supplementary Table S1, some specimens with low infectivity ingested the same amount of blood as those that had a higher infection rate. The correlation analyses between the amount of blood ingested and parasitic infection were not significant for all tests (additional files 2–5; *p* > 0.05), with the exception being the correlation between *T. b. brasiliensis* and the amount of blood ingested on the 20th day after the blood meal.

## 4. Discussion

In the literature, few studies have addressed the susceptibility of natural or experimental hybrids of triatomines to infection by *T. cruzi*. Therefore, it is extremely important to understand how a particular strain of this etiologic agent can interact with the triatomine vector species and their hybrids. In this unprecedented study, susceptibility to the *T. cruzi* strain 0354 (TcI) infection was evaluated by comparing *T. b. brasiliensis*, *T. juazeirensis*, and their reciprocal experimental hybrids. In our experiment, under laboratory conditions, it was revealed that the hybrids of the *T. brasiliensis* complex are able to be infected by *T. cruzi*, and they developed the infective forms in the rectum in higher percentages than their parentals. It is important to stress that in some cases, hybrids may be sterile, which would reduce their epidemiological importance. However, this is not the case for the hybrids of the *T. brasiliensis* complex, specifically those between *T. b. brasiliensis* and *T. juazeirensis,* since their fertility was already demonstrated until the F3 phase under laboratory conditions [21]. In addition to this, it was suggested more recently that the species’ sexual choice is not always conspecific but can increase genetic variability, which emphasizes the importance of better understanding the hybrids’ vector competence and capacity [52]. We also want to stress that the parentals, *T. b brasiliensis* and *T. juazeirensis*, play a relevant role as vectors of the etiologic agent of Chagas disease in several states of the northeast region of Brazil [27,28,29,30,31,32,36,37,53].

The host–parasite relationship drives triatomines to transmit *T. cruzi* to susceptible hosts, which involves several factors such as the morphogenesis process of the parasite [54]; the susceptibility of the insect vector to the parasite strain [55]; the average time between blood-feeding and infective defecation, which occurs when the triatomine is still in direct contact with the host’s skin; and the number of blood meals at each stage during the insect developmental cycle [56]. These characteristics provide important information that enables us to evaluate the vector capacity of the triatomine species [57], which covers several biological, ecological, and behavioral parameters of the insect; also, we can evaluate their vector competence, which is estimated as the proportion of individuals susceptible to the etiological agent within the population [58].

In our experiment, it was found that the epimastigote form was prevalent in all compartments of the digestive tract in both the hybrids and the parentals. This can be corroborated with previous findings where *T. infestans* was infected with the Y strain, in which epimastigotes, and occasionally amastigotes and metacyclic forms, were found in the intestine [59].

According to a previous analysis of the interaction of a *T. cruzi* strain with a particular vector species, the proportionality of the numbers of epimastigotes, spheromastigotes, and metacyclic trypomastigotes in the vector digestive tract can be modified according to several aspects, including the particularities of each specimen [50]. Our results showed that the populations of epimastigotes and transitional forms in the stomach of *T. juazeirensis* were significantly lower either in the hybrids or in *T. b. brasiliensis*. This difference in the proportion of these *T. cruzi* populations may affect the metacyclogenesis, which appears to be vector-dependent [40].

The decrease in the epimastigote population in the rectum and the increase in the metacyclic population in the gut are expected events in vector species susceptible to *T. cruzi* infection, such as *Rhodnius neglectus* Lent, 1954, *Rhodnius Prolixus* Stal, 1859, and *Panstrongylus megistus* (Burmeister, 1835), when infected with the Y strain [40]. In the present experiment, a higher prevalence of epimastigotes was observed in all insects’ gut compartments. The trypomastigotes had lower numbers over the experiment compared to the other forms; however, this form was recorded in significantly higher percentages in the hybrids’ rectums. An experimental infection carried out with members of the *T. brasiliensis* complex using the 913 strain demonstrated that the development of the parasite was similar in all vectors. However, mice infected with the 913 strain from *T. b. macromelasoma* (a subspecies with a hybrid origin) [23,38] had higher rates on the 20th day after infection [43]. In the present study, it was observed the experimental hybrids showed a decrease in the parasite population (in the case of Hjb) after that period (the 20th day).

Transitional forms were also distributed throughout the insects’ guts and were present in all observation periods, but their peak density oscillated over different days and groups. The presence of transitional forms is necessary for parasite development because the transformation from the epimastigote into the metacyclic form leads to an indeterminate form [40]. The speed at which this transformation occurs depends on the nature of the *T. cruzi* strain since the faster the strain can perform this process, the better the multiplication conditions will be for establishing an infection, and higher production of metacyclic trypomastigotes will also occur [54].

An experiment performed with *T. infestans* showed a tendency to overcome infection with the Y strain, whereas *P. megistus* was the species most susceptible to infection. This latter species had a better interaction with the Y strain, such that it continued to present with infection over a period of time [60]. In our experiment, the analysis of strain 0354 demonstrated that it is able to interact with *T. b. brasiliensis* and *T. juazeirensis*, especially with the hybrids. Sometimes, the strains fail to complete their life cycle, i.e., they fail to infect the digestive tract of the insect or present with low levels of infection [48]. In a previous study, for example, the infectivity of strain 0354 showed different behavior in *T. infestans*. The epimastigote form was prevalent throughout the digestive tract until the 20th day after infection, but on the 30th day, the trypomastigote metacyclic form was most frequent, especially in the rectum; this was different in the hybrids and parentals, in which the epimastigote form was more prevalent during all observation periods. This suggests that the development of strain 0354 in the *T. brasiliensis* species complex and hybrids was slower than in *T. infestans*. In analyzing the development of *T. cruzi* in the insect digestive tract, we should consider the digestive tract as a series of micro-habitats in which the parasite interacts with different factors that can modulate its development, such as the amount of blood ingested, hemolytic factors, the perimicrovillar membrane, and the insect’s innate immune system [40,41,42,61].

*Triatoma b. brasiliensis* presents with high rates of infection in nature [31,32]. On the contrary, the present study, it demonstrated a low percentage of metacyclic forms. Other studies showed that other species that are also often naturally found to have high rates of infection presented with low percentages under laboratory conditions [40].

The results from *T. juazeirensis* showed a slower process of metacyclogenesis since the metacyclic trypomastigote percentages were the lowest ones. Like the hybrids and *T. b. brasiliensis,* this species showed an ability to host and maintain the parasite. Nonetheless, *T. juazeirensis* is often found to have a low rate of natural infection when compared to the other species, as already mentioned in the literature [31,32,34,53].

For the insects to become infected with the parasite, they need to ingest an infective blood meal. Some studies have suggested that for high levels of infection, the insects would need to ingest large amounts of blood infected with the parasite. Nevertheless, it is also known that the persistence of the parasite in the insect will depend on several factors relating to the insect’s immune system [15]. In our study, insects with high levels of infection (~49 × 10^5^) ingested the same or lower amounts of blood (~220 mg) than the specimens with lower levels of infection (~4 × 10^5^). Moreover, specimens that ingested very small amounts of blood (e.g., 91 mg) had a sufficient quantity of parasites to initiate and maintain the infection in their digestive tract for up to 30 days after feeding. Another study evaluating the susceptibility of four species of triatomines to infection with the Y strain also found the amount of blood ingested did not have any influence on the level of infection presented by the insects [55].

All of the insect groups analyzed were susceptible to infection with *T. cruzi* strain 0354, but the hybrids maintained greater stability of infectivity, presenting with higher numbers of metacyclic trypomastigotes in the rectum throughout the experiment and across all observation periods. This means that the hybrids could be more susceptible to infection than the parentals. This difference between parentals and hybrids might be the result of the distinct efficiency of qualitative interactions between the strain and the vector. In comparison with *T. infestans*, *P. megistus* also became infected with the *T. cruzi* Y strain more efficiently, thus demonstrating higher numbers of positive insects, increased positivity over time, and a higher maintenance rate of infection [60].

Small intraspecific differences in the susceptibility among triatomines infected with the same strain of *T. cruzi* have been proven to occur. A group of *T. infestans* reared in the same colony showed different rates of infection in comparison with a group of specimens newly captured from the natural environment [62]. This may explain why *T. b. brasiliensis* initially presented with infection with strain 0354 at a lower rate than that of the hybrids. Additionally, this vector is known for its high infection rates in natural environments, and it was expected to have higher levels than the other groups [27,31,32].

The natural ecotope of the insects also needs to be considered since the specimens used in these experiments were all reared under laboratory conditions. Some studies have emphasized the superiority of wild vectors over domestic ones for studying *T. cruzi* infection [61], such as *T. infestans* and *R. neglectus* infected with the Y strain, which had a lower infection rate than *Dipetalogaster maxima* (Uhler, 1894) and *Triatoma matogrossensis* Leite & Barbosa, 1953, and which are typically wild species that invade domiciles [55].

The results reported here open up new avenues to be explored and can be applied for triatomine control. The interaction between *T. cruzi* and triatomines is a complex issue; therefore, the higher susceptibility to *T. cruzi* observed in the hybrid specimens could be associated with specific factors that must be deeply analyzed and experimentally tested. Among the possibilities for future related studies that compare triatomine hybrids and parentals, studying the gut components and microbiota, as stressed by Fuentes–Vicente [20], could help to improve the triatomine control program and reduce the use of insecticides.

## 5. Conclusions

In the present study, the 0354 *T. cruzi* strain was shown to be able to develop and maintain its cycle in the digestive tract of *T. b. brasiliensis* and *T. juazeirensis* as well as in that of their experimental hybrids. The obtained results, in addition to recent findings that showed the presence of natural hybrids in areas where different species of the *T. brasiliensis* complex are circulating, contribute to the consideration of the potential participation of these hybrids in the natural transmission cycle of *T. cruzi*. The hybrids showed greater parasite distributional homogeneity for up to 30 days after infection. However, further analysis is required to determine whether *T. b. brasiliensis* might present with more infective forms in the rectum, even though its development initially was slower. The same analyses would be necessary for *T. juazeirensis*; nevertheless, this species showed greater numbers of negative specimens than the other analyzed groups, and therefore, its vector competence seems to be less effective than that observed for *T. b. brasiliensis* and the hybrids.

The present study points out that the hybrids, as potential vectors of *T. cruzi*, are able to transmit the etiological agent in the natural hybrid zones where *T. b. brasiliensis* and *T. juazeirensis* can be found and enable us to understand the dynamics of some of the mechanisms that occur in the development of strain 0354. Further analysis of the susceptibility of these insects to different *T. cruzi* strains, as well as the infection of these hybrids in the natural environment, needs to be conducted.

## Figures and Tables

**Table 1:** Data on the localities of the founder species of the colonies.

Species	State	Municipality	Date	Geographic Coordinates
*T. b. brasiliensis*	RN	Caicó	12 May 2011	06°27′ S 37°05′ W
*T. juazeirensis*	BA	Curaça	24 November 2013	09°12′ S 39°83′ W

**Table 2:** Percentages of different forms of *Trypanosoma cruzi* found in different compartments of the digestive tube (stomach, intestine, and rectum) on the 10th, 20th, and 30th days after infection in *Triatoma b. brasiliensis*, *Triatoma juazeirensis*, and their hybrids Hbj (♀ *T. b. brasiliensis* × ♂ *T. juazeirensis*) and Hjb (♀ *T. juazeirensis* × ♂ *T. b. brasiliensis*) throughout the experiment.

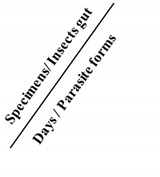		Stomach			Intestine			Rectum		
		Epimastigotes	Transition forms	Trypomastigotes	Epimastigotes	Transition forms	Trypomastigotes	Epimastigotes	Transition forms	Trypomastigotes
Hybrids Hbj	10	68.59	7.28	5.29	10.3	1.22	2.58	3.88	0.60	0.26
	20	49.54	7.43	8.02	18.02	1.86	5.64	7.17	0.78	1.53
	30	6.53	4.02	1.51	52.54	5.32	3.67	20.94	2.46	3.01
Hybrids Hjb	10	75.38	5.54	4.85	8.89	0.91	0.75	3.08	0.25	0.35
	20	56.74	9.44	7.33	12.91	1.74	2.11	6.03	0.70	3.01
	30	0.45	0.42	0	60.25	11.27	11.78	14.37	0.62	0.84
*T. juazeirensis*	10	62.18	7.79	0.94	26.62	1.85	0.36	0.24	0.01	0
	20	3.06	0.87	0	75.30	11.11	0.04	7.87	1.53	0.22
	30	15.73	4.50	0.05	61.58	6.57	3.86	6.49	0.72	0.50
*T. b. brasiliensis*	10	78.39	5.03	1.41	13.17	0.90	0	1.06	0.04	0
	20	20.70	5.70	0.81	55.91	7.17	0.08	8.15	1.34	0.12
	30	12.27	1.15	0	56.28	7.59	2.36	16.23	2.65	1.47

**Table 3:** Relative percentages of different forms of *Trypanosoma cruzi* found in different compartments of the digestive tube (stomach, intestine, and rectum) on the 10th, 20th, and 30th days after infection in *Triatoma b. brasiliensis*, *Triatoma juazeirensis*, and their hybrids Hbj (♀ *T. b. brasiliensis* × ♂ *T. juazeirensis*) and Hjb (♀ *T. juazeirensis* × ♂ *T. b. brasiliensis*).

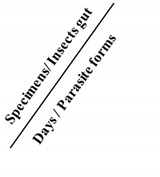		Stomach			Intestine			Rectum		
		Epimastigotes	Transition forms	Trypomastigotes	Epimastigotes	Transition forms	Trypomastigotes	Epimastigotes	Transition forms	Trypomastigotes
Hybrids Hbj	10	84.5	8.7	6.5	73.1	8.6	18.3	81.8	12.7	5.5
	20	76.2	11.4	12.3	70.6	7.3	22.1	75.6	8.2	16.2
	30	54.2	33.3	12.5	85.4	8.7	6.0	79.0	9.3	11.4
Hybrids Hjb	10	87.9	6.5	5.7	84.3	8,6	7.1	83.7	6.7	9.6
	20	77.2	12.8	10.0	77.0	10.4	12.6	62.0	7.2	30.9
	30	51.6	48.4	0.0	72.3	13.5	14.1	90.8	3.9	5.3
*T. juazeirensis*	10	87.7	11.0	1.3	92.3	6.4	1.3	94.7	5.3	0
	20	77.8	22.2	0	87.1	12.8	0.1	81.8	15.9	2.3
	30	77.6	22.2	0.3	85.5	9.1	5.4	84.1	9.4	6.5
*T. b. brasiliensis*	10	92.4	5.9	1.7	93.6	6.4	0	96.0	4.0	0
	20	76.0	21.0	3.0	88.5	11.4	0.1	84.7	14.0	1.3
	30	91.5	8.5	0	85.0	11.5	3.6	79.7	13.0	7.2

## Data Availability

The datasets supporting the conclusions of this article are included within the article and its additional files.

## Supplementary Materials

The following supporting information can be downloaded at: https://www.mdpi.com/article/10.3390/microorganisms11122850/s1, Supplementary Table S1. Volume of blood (mg) ingested and parasitic population density per specimen on the 10th, 20th, and 30th days after the blood meal. Supplementary Table S2. Number of *T. cruzi* parasites in each segment of the digestive tract individually displayed on a hemocytometer to count the distinct forms: epimastigotes, transitional forms, and trypomastigotes.

## Abbreviations

Hbj—F1 hybrids from crossing ♀ *T. b. brasiliensis* × ♂ *T. juazeirensis*; Hjb—F1 hybrids from crossing ♀ *T. juazeirensis* × ♂ *T. b. brasiliensis*; BA_Bahia; RN_Rio Grande do Norte; COLTRYP—Collection of Trypanosoma of Wild and Domestic Mammals and Vectors, from the Oswaldo Cruz Institute; PCR—polymerase chain reaction; NNN—biphasic culture medium (MacNeal, Novy, and Nicolle); LIT—liver infusion tryptose culture medium; *v*/*v*—volume/volume; PBS—phosphate-buffered saline.
