# Supplementary material for: Experimental Hybrids of the Triatoma brasiliensis Species Complex Show Higher Susceptibility to the Trypanosoma cruzi Infection Than Their Parentals
Source: Microorganisms. 2023 Nov 24;11(12):2850. doi: 10.3390/microorganisms11122850 (PMC10745812; doi:10.3390/microorganisms11122850)
Supplement: Supplementary file 1 [file microorganisms-11-02850-s001.zip › microorganisms-2685243-supplementary.pdf]

Supplementary Table S1- Blood intake(mg) and parasitic density found in the specimens examined on the 10th, 20th and 30th days after the T.cruzi infection

	Days after the infection					
	10		20		30	
Specimens	Weight (mg)	Parasites	Weight (mg)	Parasites	Weight(mg)	Parasites
HybridsHbj	230	$14x10^5$	200	$7x10^5$	157	$1x10^5$
	116	$27x10^5$	95	$8x10^5$	113	$2x10^5$
	227	$21x10^5$	162	$7x10^5$	91	$4x10^5$
	197	$20x10^5$	141	$21x10^5$	127	$9x10^5$
	344	$20x10^5$	216	$5x10^5$	128	$7x10^5$
	107	$24x10^5$	364	$5x10^5$	225	$4x10^5$
	150	$36x10^5$	283	$21x10^5$	108	$10x10^5$
	391	$23x10^5$	332	$6x10^5$	75	$3x10^5$
	296	$22x10^5$	117	$24x10^5$	162	$2x10^5$
	154	$14x10^5$	228	$2x10^5$	111	$7x10^5$
HybridsHjb	480	$7x10^5$	110	$10x10^5$	177	$0,5x10^5$
	334	$30x10^5$	127	$23x10^5$	123	$13x10^5$
	121	$18x10^5$	535	$12x10^5$	111	$18x10^5$
	235	$15x10^5$	125	$0,8x10^5$	155	$10x10^5$
	262	$14x10^5$	291	$1x10^5$	196	$1x10^5$
	116	$11x10^5$	108	$7x10^5$	65	$8x10^5$
	134	$9x10^5$	48	$11x10^5$	209	$3x10^5$
	124	$14x10^5$	153	$28x10^5$	228	$2x10^5$
	257	$27x10^5$	438	$10x10^5$	20	$0,1x10^5$
	242	$17x10^5$	302	$33x10^5$	155	$32x10^5$
Triatoma juazeirensis	43	$11x10^5$	83	$12x10^5$	205	$12x10^5$
	123	0	94	$1x10^5$	396	$20x10^5$
	146	$0,3x10^5$	109	$2x10^5$	102	$24x10^5$
	163	$2x10^5$	140	$2x10^5$	177	$82x10^5$
	164	$18x10^5$	141	$9x10^5$	89	$0,8x10^5$
	239	$43x10^5$	159	$17x10^5$	257	$2x10^5$
	295	$6x10^5$	218	$6x10^5$	149	$3x10^5$
	328	$0,6x10^5$	278	$2x10^5$	152	$47x10^5$
	390	$9x10^5$	328	$2x10^5$	160	$16x10^5$
	213	$97x10^5$	336	$2x10^5$	142	$28x10^5$
Triatomab. brasiliensis	220	$49x10^5$	707	$27x10^5$	241	$4x10^5$
	209	$38x10^5$	345	$6x10^5$	307	$5x10^5$
	140	$80x10^5$	297	$3x10^5$	363	$7x10^5$
	75	$48x10^5$	286	$4x10^5$	128	$3x10^5$
	202	$50x10^5$	410	$6x10^5$	170	$10x10^5$
	311	$43x10^5$	184	$1x10^5$	383	$3x10^5$
	100	$10x10^5$	410	$5x10^5$	480	$8x10^5$
	101	$36x10^5$	59	$2x10^5$	375	$6x10^5$
	92	$28x10^5$	125	$4x10^5$	297	$25x10^5$
	140	$28x10^5$	219	$2x10^5$	320	$6x10^5$

Supplementary Table S2-

Group	Numbers of parasites<br>epimastigotes	transitional	tripomastigotes	day	segment
HJB -1	337500	50000	62500	**10**	stomach
HJB -2	2725000	87500	0	10	stomach
HJB-3	1400000	137500	50000	10	stomach
HJB-4	1037500	212500	75000	10	stomach
HJB-5	1100000	87500	62500	10	stomach
HJB-6	787500	62500	87500	10	stomach
HJB-7	525000	25000	100000	10	stomach
HJB-8	975000	62500	50000	10	stomach
HJB-9	2100000	112500	225000	10	stomach
HJB-10	1262500	62500	75000	10	stomach
HBJ-1	712500	200000	0	10	stomach
HBJ-2	1875000	425000	37500	10	stomach
HBJ-3	1300000	125000	125000	10	stomach
HBJ-4	1187500	175000	87500	10	stomach
HBJ-5	1487500	87500	62500	10	stomach
HBJ-6	1475000	62500	137500	10	stomach
HBJ-7	2750000	187500	425000	10	stomach
HBJ-8	1537500	150000	125000	10	stomach
HBJ-9	1762500	150000	125000	10	stomach
HBJ-10	987500	37500	37500	10	stomach
BRA-1	3825000	225000	0	10	stomach
BRA-2	3225000	200000	0	10	stomach
BRA-3	0	0	0	10	stomach
BRA-4	2900000	175000	50000	10	stomach
BRA-5	4250000	375000	175000	10	stomach
BRA-6	3900000	150000	175000	10	stomach
BRA-7	825000	100000	0	10	stomach
BRA-8	2975000	200000	0	10	stomach
BRA-9	2400000	100000	0	10	stomach
BRA-10	2175000	175000	75000	10	stomach
JUAZ-1	0	0	0	10	stomach
JUAZ-2	0	0	0	10	stomach
JUAZ-3	0	0	0	10	stomach
JUAZ-4	150000	50000	0	10	stomach
JUAZ-5	825000	150000	0	10	stomach
JUAZ-6	1575000	275000	0	10	stomach
JUAZ-7	0	0	0	10	stomach
JUAZ-8	0	0	0	10	stomach
JUAZ-9	425000	100000	50000	10	stomach
JUAZ-10	8600000	875000	125000	10	stomach

HJB -1	167500	12500	20000	10	intestine
HJB -2	72500	5000	5000	10	intestine
HJB-3	62500	7500	7500	10	intestine
HJB-4	187500	22500	7500	10	intestine
HJB-5	30000	2500	2500	10	intestine
HJB-6	97500	17500	15000	10	intestine
HJB-7	215000	30000	25000	10	intestine
HJB-8	135000	12500	12500	10	intestine
HJB-9	185000	22500	17500	10	intestine
HJB-10	292500	15000	10000	10	intestine
HBJ-1	375000	42500	0	10	intestine
HBJ-2	105000	25000	17500	10	intestine
HBJ-3	302500	87500	137500	10	intestine
HBJ-4	292500	35000	155000	10	intestine
HBJ-5	282500	17500	62500	10	intestine
HBJ-6	262500	10000	65000	10	intestine
HBJ-7	132500	7500	42500	10	intestine
HBJ-8	160000	0	32500	10	intestine
HBJ-9	92500	27500	12500	10	intestine
HBJ-10	260000	15000	42500	10	intestine
BRA-1	720000	45000	0	10	intestine
BRA-2	325000	25000	0	10	intestine
BRA-3	750000	50000	0	10	intestine
BRA-4	1412500	60000	0	10	intestine
BRA-5	147500	17500	0	10	intestine
BRA-6	50000	5000	0	10	intestine
BRA-7	57500	10000	0	10	intestine
BRA-8	307500	37500	0	10	intestine
BRA-9	312500	17500	0	10	intestine
BRA-10	365000	35000	0	10	intestine
JUAZ-1	912500	125000	35000	10	intestine
JUAZ-2	0	0	0	10	intestine
JUAZ-3	0	0	0	10	intestine
JUAZ-4	15000	2500	0	10	intestine
JUAZ-5	750000	50000	25000	10	intestine
JUAZ-6	2325000	75000	0	10	intestine
JUAZ-7	530000	57500	2500	10	intestine
JUAZ-8	52500	10000	0	10	intestine
JUAZ-9	307500	20000	2500	10	intestine
JUAZ-10	62500	5000	2500	10	intestine
HJB -1	82500	0	7500	10	rectum
HJB -2	80000	5000	12500	10	rectum
HJB-3	77500	5000	7500	10	rectum
HJB-4	2500	0	0	10	rectum

HJB-5	92500	10000	10000	10	rectum
HJB-6	7500	0	7500	10	rectum
HJB-7	10000	2500	0	10	rectum
HJB-8	140000	12500	12500	10	rectum
HJB-9	0	0	0	10	rectum
HJB-10	7500	5000	0	10	rectum
HBJ-1	32500	10000	0	10	rectum
HBJ-2	160000	32500	7500	10	rectum
HBJ-3	10000	0	5000	10	rectum
HBJ-4	22500	0	0	10	rectum
HBJ-5	37500	2500	7500	10	rectum
HBJ-6	320000	15000	17500	10	rectum
HBJ-7	52500	2500	7500	10	rectum
HBJ-8	205000	70000	7500	10	rectum
HBJ-9	0	0	0	10	rectum
HBJ-10	12500	0	5000	10	rectum
BRA-1	47500	2500	0	10	rectum
BRA-2	0	0	0	10	rectum
BRA-3	2500	0	0	10	rectum
BRA-4	225000	0	0	10	rectum
BRA-5	0	0	0	10	rectum
BRA-6	10000	0	0	10	rectum
BRA-7	15000	7500	0	10	rectum
BRA-8	52500	5000	0	10	rectum
BRA-9	0	0	0	10	rectum
BRA-10	5000	0	0	10	rectum
JUAZ-1	0	0	0	10	rectum
JUAZ-2	0	0	0	10	rectum
JUAZ-3	27500	2500	0	10	rectum
JUAZ-4	0	0	0	10	rectum
JUAZ-5	0	0	0	10	rectum
JUAZ-6	0	0	0	10	rectum
JUAZ-7	7500	0	0	10	rectum
JUAZ-8	0	0	0	10	rectum
JUAZ-9	0	0	0	10	rectum
JUAZ-10	10000	0	0	10	rectum
HJB -1	537500	162500	50000	**20**	stomach
HJB -2	1662500	62500	212500	20	stomach
HJB-3	712500	112500	62500	20	stomach
HJB-4	50000	0	0	20	stomach
HJB-5	25000	12500	0	20	stomach
HJB-6	112500	162500	0	20	stomach
HJB-7	812500	112500	37500	20	stomach
HJB-8	1250000	425000	400000	20	stomach

HJB-9	400000	212500	212500	20	stomach
HJB-10	2175000	25000	25000	20	stomach
HBJ-1	362500	87500	0	20	stomach
HBJ-2	150000	62500	0	20	stomach
HBJ-3	112500	0	12500	20	stomach
HBJ-4	1287500	175000	400000	20	stomach
HBJ-5	62500	25000	0	20	stomach
HBJ-6	200000	50000	25000	20	stomach
HBJ-7	1625000	112500	125000	20	stomach
HBJ-8	200000	87500	12500	20	stomach
HBJ-9	1250000	187500	275000	20	stomach
HBJ-10	0	0	0	20	stomach
BRA-1	375000	175000	25000	20	stomach
BRA-2	7500	2500	0	20	stomach
BRA-3	7500	2500	0	20	stomach
BRA-4	112500	22500	0	20	stomach
BRA-5	417500	32500	25000	20	stomach
BRA-6	0	0	0	20	stomach
BRA-7	150000	67500	0	20	stomach
BRA-8	27500	2500	0	20	stomach
BRA-9	172500	45000	0	20	stomach
BRA-10	0	0	0	20	stomach
JUAZ-1	37500	0	0	20	stomach
JUAZ-2	0	0	0	20	stomach
JUAZ-3	0	0	0	20	stomach
JUAZ-4	0	0	0	20	stomach
JUAZ-5	112500	50000	0	20	stomach
JUAZ-6	25000	0	0	20	stomach
JUAZ-7	0	0	0	20	stomach
JUAZ-8	0	0	0	20	stomach
JUAZ-9	0	0	0	20	stomach
JUAZ-10	0	0	0	20	stomach
HJB -1	250000	12500	25000	20	intestine
HJB -2	245000	12500	87500	20	intestine
HJB-3	162500	45000	12500	20	intestine
HJB-4	12500	2500	0	20	intestine
HJB-5	47500	2500	10000	20	intestine
HJB-6	332500	102500	10000	20	intestine
HJB-7	97500	2500	27500	20	intestine
HJB-8	377500	37500	50000	20	intestine
HJB-9	77500	5000	10000	20	intestine
HJB-10	157500	15000	55000	20	intestine
HBJ-1	202500	17500	12500	20	intestine
HBJ-2	205000	12500	50000	20	intestine

HBJ-3	162500	20000	67500	20	intestine
HBJ-4	150000	7500	17500	20	intestine
HBJ-5	262500	12500	25000	20	intestine
HBJ-6	117500	37500	35000	20	intestine
HBJ-7	180000	20000	27500	20	intestine
HBJ-8	215000	17500	27500	20	intestine
HBJ-9	310000	10000	312500	20	intestine
HBJ-10	105000	42500	22500	20	intestine
BRA-1	2025000	125000	0	20	intestine
BRA-2	400000	175000	0	20	intestine
BRA-3	275000	0	0	20	intestine
BRA-4	150000	50000	0	20	intestine
BRA-5	132500	12500	0	20	intestine
BRA-6	20000	0	0	20	intestine
BRA-7	175000	30000	0	20	intestine
BRA-8	77500	12500	0	20	intestine
BRA-9	132500	27500	5000	20	intestine
BRA-10	42500	7500	0	20	intestine
JUAZ-1	775000	112500	0	20	intestine
JUAZ-2	105000	10000	2500	20	intestine
JUAZ-3	137500	25000	0	20	intestine
JUAZ-4	200000	50000	0	20	intestine
JUAZ-5	650000	150000	0	20	intestine
JUAZ-6	1550000	125000	0	20	intestine
JUAZ-7	450000	100000	0	20	intestine
JUAZ-8	187500	0	0	20	intestine
JUAZ-9	175000	50000	0	20	intestine
JUAZ-10	75000	12500	0	20	intestine
HJB -1	0	0	0	20	rectum
HJB -2	27500	0	0	20	rectum
HJB-3	37500	10000	10000	20	rectum
HJB-4	15000	2500	0	20	rectum
HJB-5	27500	2500	10000	20	rectum
HJB-6	2500	7500	0	20	rectum
HJB-7	7500	0	12500	20	rectum
HJB-8	197500	27500	12500	20	rectum
HJB-9	47500	10000	12500	20	rectum
HJB-10	460000	35000	352500	20	rectum
HBJ-1	35000	5000	10000	20	rectum
HBJ-2	222500	20000	35000	20	rectum
HBJ-3	247500	22500	75000	20	rectum
HBJ-4	52500	5000	0	20	rectum
HBJ-5	100000	12500	17500	20	rectum
HBJ-6	27500	5000	20000	20	rectum

HBJ-7	12500	0	5000	20	rectum
HBJ-8	57500	10000	0	20	rectum
HBJ-9	5000	2500	0	20	rectum
HBJ-10	0	0	0	20	rectum
BRA-1	12500	0	0	20	rectum
BRA-2	5000	2500	0	20	rectum
BRA-3	22500	0	0	20	rectum
BRA-4	17500	7500	0	20	rectum
BRA-5	27500	2500	0	20	rectum
BRA-6	87500	10000	0	20	rectum
BRA-7	80000	10000	0	20	rectum
BRA-8	77500	12500	0	20	rectum
BRA-9	27500	10000	0	20	rectum
BRA-10	142500	27500	7500	20	rectum
JUAZ-1	200000	35000	12500	20	rectum
JUAZ-2	30000	5000	0	20	rectum
JUAZ-3	25000	0	0	20	rectum
JUAZ-4	0	0	0	20	rectum
JUAZ-5	0	0	0	20	rectum
JUAZ-6	0	0	0	20	rectum
JUAZ-7	70000	12500	0	20	rectum
JUAZ-8	55000	10000	0	20	rectum
JUAZ-9	20000	7500	0	20	rectum
JUAZ-10	50000	17500	0	20	rectum
HJB -1	0	0	0	**30**	stomach
HJB -2	0	0	0	30	stomach
HJB-3	0	0	0	30	stomach
HJB-4	0	0	0	30	stomach
HJB-5	7500	0	0	30	stomach
HJB-6	5000	0	0	30	stomach
HJB-7	2500	0	0	30	stomach
HJB-8	12500	0	0	30	stomach
HJB-9	0	0	0	30	stomach
HJB-10	12500	37500	0	30	stomach
HBJ-1	0	0	0	30	stomach
HBJ-2	0	0	0	30	stomach
HBJ-3	75000	150000	25000	30	stomach
HBJ-4	0	0	0	30	stomach
HBJ-5	150000	25000	0	30	stomach
HBJ-6	50000	0	0	30	stomach
HBJ-7	50000	0	25000	30	stomach
HBJ-8	0	0	0	30	stomach
HBJ-9	0	25000	0	30	stomach
HBJ-10	0	0	25000	30	stomach

BRA-1	0	0	0	30	stomach
BRA-2	0	0	0	30	stomach
BRA-3	112500	25000	0	30	stomach
BRA-4	0	0	0	30	stomach
BRA-5	150000	37500	0	30	stomach
BRA-6	0	0	0	30	stomach
BRA-7	75000	0	0	30	stomach
BRA-8	0	0	0	30	stomach
BRA-9	550000	25000	0	30	stomach
BRA-10	50000	0	0	30	stomach
JUAZ-1	15000	5000	0	30	stomach
JUAZ-2	1225000	400000	0	30	stomach
JUAZ-3	1625000	575000	0	30	stomach
JUAZ-4	575000	75000	0	30	stomach
JUAZ-5	37500	0	0	30	stomach
JUAZ-6	125000	0	0	30	stomach
JUAZ-7	12500	0	0	30	stomach
JUAZ-8	37500	0	12500	30	stomach
JUAZ-9	12500	0	0	30	stomach
JUAZ-10	25000	0	0	30	stomach
HJB -1	15000	15000	0	30	intestine
HJB -2	235000	32500	35000	30	intestine
HJB-3	642500	475000	725000	30	intestine
HJB-4	337500	275000	250000	30	intestine
HJB-5	100000	0	0	30	intestine
HJB-6	717500	22500	25000	30	intestine
HJB-7	155000	7500	12500	30	intestine
HJB-8	100000	50000	0	30	intestine
HJB-9	5000	0	0	30	intestine
HJB-10	3050000	125000	0	30	intestine
HBJ-1	15000	0	5000	30	intestine
HBJ-2	7500	5000	0	30	intestine
HBJ-3	160000	17500	5000	30	intestine
HBJ-4	447500	15000	32500	30	intestine
HBJ-5	410000	85000	20000	30	intestine
HBJ-6	55000	7500	7500	30	intestine
HBJ-7	700000	25000	50000	30	intestine
HBJ-8	200000	22500	7500	30	intestine
HBJ-9	145000	12500	5000	30	intestine
HBJ-10	475000	75000	50000	30	intestine
BRA-1	32500	7500	2500	30	intestine
BRA-2	357500	52500	0	30	intestine
BRA-3	400000	75000	25000	30	intestine
BRA-4	157500	15000	15000	30	intestine

BRA-5	637500	87500	0	30	intestine
BRA-6	155000	35000	0	30	intestine
BRA-7	525000	75000	50000	30	intestine
BRA-8	247500	20000	37500	30	intestine
BRA-9	1525000	175000	50000	30	intestine
BRA-10	262500	37500	0	30	intestine
JUAZ-1	77500	10000	0	30	intestine
JUAZ-2	295000	75000	0	30	intestine
JUAZ-3	177500	27500	2500	30	intestine
JUAZ-4	6725000	125000	700000	30	intestine
JUAZ-5	17500	5000	0	30	intestine
JUAZ-6	5000	0	2500	30	intestine
JUAZ-7	225000	0	0	30	intestine
JUAZ-8	3150000	975000	150000	30	intestine
JUAZ-9	1550000	50000	0	30	intestine
JUAZ-10	2225000	275000	50000	30	intestine
HJB -1	15000	2500	0	30	rectum
HJB -2	985000	0	20000	30	rectum
HJB-3	0	0	0	30	rectum
HJB-4	80000	40000	5000	30	rectum
HJB-5	0	2500	0	30	rectum
HJB-6	32500	5000	35000	30	rectum
HJB-7	137500	0	12500	30	rectum
HJB-8	15000	0	2500	30	rectum
HJB-9	7500	5000	0	30	rectum
HJB-10	5000	0	0	30	rectum
HBJ-1	45000	15000	35000	30	rectum
HBJ-2	177500	20000	22500	30	rectum
HBJ-3	0	0	0	30	rectum
HBJ-4	365000	20000	27500	30	rectum
HBJ-5	10000	0	2500	30	rectum
HBJ-6	210000	35000	15000	30	rectum
HBJ-7	117500	15000	5000	30	rectum
HBJ-8	30000	2500	17500	30	rectum
HBJ-9	0	0	0	30	rectum
HBJ-10	87500	15000	25000	30	rectum
BRA-1	275000	30000	32500	30	rectum
BRA-2	32500	7500	5000	30	rectum
BRA-3	100000	0	7500	30	rectum
BRA-4	72500	0	5000	30	rectum
BRA-5	65000	20000	0	30	rectum
BRA-6	90000	12500	0	30	rectum
BRA-7	62500	12500	27500	30	rectum
BRA-8	250000	75000	0	30	rectum

BRA-9	122500	27500	12500	30	rectum
BRA-10	170000	17500	22500	30	rectum
JUAZ-1	847500	120000	105000	30	rectum
JUAZ-2	0	0	0	30	rectum
JUAZ-3	0	0	0	30	rectum
JUAZ-4	17500	0	0	30	rectum
JUAZ-5	20000	2500	2500	30	rectum
JUAZ-6	27500	0	0	30	rectum
JUAZ-7	40000	0	0	30	rectum
JUAZ-8	342500	40000	5000	30	rectum
JUAZ-9	32500	2500	0	30	rectum
JUAZ-10	195000	5000	5000	30	rectum
